# Association between socioeconomic factors at diagnosis and survival in breast cancer: A population‐based study

**DOI:** 10.1002/cam4.2842

**Published:** 2020-01-20

**Authors:** Peng Ji, Yue Gong, Chang‐Chuan Jiang, Xin Hu, Gen‐Hong Di, Zhi‐Ming Shao

**Affiliations:** ^1^ Department of Breast Surgery Key Laboratory of Breast Cancer in Shanghai Fudan University Shanghai Cancer Center Fudan University Shanghai China; ^2^ Department of Oncology Shanghai Medical College Fudan University Shanghai China; ^3^ Department of Medicine Icahn School of Medicine at Mount Sinai Mt Sina St. Luke's and Mt Sinai West Hospital New York NY USA; ^4^ Institutes of Biomedical Science Fudan University Shanghai China

**Keywords:** breast cancer, nomogram, SEER, socioeconomic, survival

## Abstract

**Background:**

The associations between socioeconomic statuses and survival outcomes of breast cancer remain unclear. No model has included both histological and socioeconomic factors to predict the survival of breast cancer. This study was designed to develop nomograms to predict breast cancer–specific survival (BCSS) and overall survival (OS) with consideration of socioeconomic factors for breast cancer patients.

**Materials and methods:**

We included a total of 207 749 female patients, diagnosed with malignant breast cancer between 2007 and 2012 from the Surveillance, Epidemiology, and End Results database. BCSS and OS were evaluated with Gray's test and log‐rank tests, respectively. Marital statuses, insurance statuses, residence, median household income, poverty rate, unemployment rate, and education level were included as socioeconomic factors in univariate and multivariate Cox regression analyses. Clinicopathological factors and socioeconomic factors were integrated to construct nomograms. Calibration plots and concordance indexes (C‐indexes) were used to evaluate the accuracy and discrimination of the models.

**Results:**

Four and three socioeconomic factors were involved in constructing the nomograms for 3‐, 5‐, and 7‐year BCSS and OS, respectively. The C‐indexes of the final nomograms were higher than those of the TNM staging system for predicting BCSS (0.835 vs 0.782; *P* < .001) and OS (0.773 vs 0.676; *P* < .001). The performance of the nomograms for predicting OS was significantly lower when excluding socioeconomic factors (*P* < .001).

**Conclusion:**

These findings may highlight the importance of developing health‐related policies and the necessity of targeted social support‐based interventions for high‐risk patients.

## Graphical abstract

The findings from this population‐based study identify important socioeconomic factors at diagnosis, namely insurance status, marital status, median household income, educational level, and residence, which are closely associated with the survival of breast cancer. A survival prediction model for breast cancer patients was developed and validated based on nomograms, and for convenience of the users, we transferred models into a user‐friendly online tool. Both doctors and patients can use this online tool for free to predict survival from perspectives of pathology and socioeconomics, which is more accurate than traditional TNM staging.

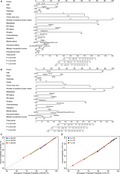

## 1 BACKGROUND

Breast cancer is a major public health concern for women worldwide. Up to 1 in 8 American women develop breast cancer during their lifetimes.^1^ However, due to genetic differences, healthcare conditions, environmental factors, and other reasons, the regional differences in the incidence and mortality of breast cancer are profound. In 2018, California ranked first among the states, with approximately 29 360 new cases, while Wyoming, ranking at the bottom, reported only 450.^2^ The death rates of breast cancer varied from 15.9 to 28.9 per 100 000 individuals in different states of the United States. In addition, racial disparity is obvious. From 2005 to 2014, despite a slight increase in the incidence among the whole population, Asian/Pacific Islander women showed an increased risk of 1.7% per year, Hispanic and black women showed an increased risk from 0.3% to 0.4% per year, and non‐Hispanic whites and American Indians/Alaska natives showed a stable trend in incidence.^1^, ^2^

Although the emergence of new drugs, early detection methods, and effective therapeutic modalities have prolonged the survival of breast cancer, regional and racial disparities are persistent. Deaths caused by breast cancer are continuously increasing in less developed regions, such as South America and Africa, partly due to limited access to healthcare. When comparing the survival of patients among different races, gaps were persistent and documented, especially in the United States, and black patients had the worst survival for all cancer types.^3^ In addition to race, socioeconomic status, which comprises insurance status, marital status, income level, education level, employment status, and other factors, was reported to have various impacts on the survival of breast cancer by affecting the stage at diagnosis and treatment compliance and adherence.^3^, ^4^, ^5^, ^6^, ^7^, ^8^, ^9^ Recently, more attention has focused on the socioeconomic determinants of breast cancer survival. Aizer et al showed that the survival benefit associated with marriage for breast cancer patients was even greater than for chemotherapy and that married patients were less likely to develop metastatic disease.^10^, ^11^ Insurance has been proven to affect the stage at diagnosis, chemotherapy initiation, adjuvant endocrine therapy adherence, and survival of breast cancer.^3^, ^12^ Although mammogram and ultrasonography are helpful in the early diagnosis of breast cancer, the popularity of screening programs largely depends on the income level and health system of a country.^12^ A person's place of residence, that is, metropolis or non‐metropolis, affects their access to screening and medical resources, while educational level has an impact on cancer awareness and adherence to treatment.^13^

However, the results of studies from different areas or different populations were not in conformity due to the complexity of socioeconomic factors. Moreover, to date, no such study constructed a survival model that includes simplified socioeconomic factors to predict the outcomes of early breast cancer. Therefore, the objective of our study was to identify the association between socioeconomic factors and survival of breast cancer among populations from 18 registries of the Surveillance, Epidemiology, and End Results (SEER) database. Furthermore, we aimed to construct a nomogram including both histology and socioeconomic factors to predict survival, which can more comprehensively improve the accuracy of predicting outcomes.

## 2 MATERIAL AND METHODS

### 2.1 Study population

We extracted data from the SEER 18 registries research database (1975‐2016) of the National Cancer Institute, which consists of 18 population‐based cancer registries and represents approximately 28% of the total population in the United States. Eligible patients were identified through SEER*Stat Version 8.3.6 (http://www.seer.cancer.gov/seerstat).^14^

We included female patients aged 18 years or older at the time of their breast cancer diagnosis between 2007 and 2012. The included patients were diagnosed before death and had histologically confirmed disease. Patients diagnosed before 2007 were not included because insurance status was not recorded in the SEER database until 2007. All variables included in the analysis had a reporting rate greater than 90%. Ineligible cases were excluded according to the following criteria: (a) prior malignancy; (b) bilateral breast cancer; (c) grade IV breast cancer; and (d) unknown or missing information on important variables, such as race, histological grade, tumor size, number of positive lymph nodes, metastasis, estrogen receptor (ER) status, progesterone receptor (PR) status, specific surgical treatment, marital status, insurance status, and survival information. After the exclusion criteria were applied, 207 749 women were eventually eligible for analysis. The flowchart of the data selection procedure is shown in Figure S1.

### 2.2 Socioeconomic factors and outcomes

Socioeconomic factors, including marital status, insurance status, residence, median household income, poverty rate, unemployment rate, and education level, were assessed in this study. The first two variables were determined at the patient level. Marital status was classified as married, single (never married), and separated/divorced/widowed, while insurance status was characterized as non‐Medicaid insured (including Medicare, military coverage, or private payers), Medicaid, and uninsured. Patients with both Medicaid and Medicare are coded as Medicaid in the SEER database and were treated as such in this analysis. Estimates of the other five types of socioeconomic status were performed at the county level and obtained from the US Census 2013‐2017 American Community Survey 5‐year data files, which were provided through the SEER*Stat software.^15^ The poverty rate was determined as the percentage of persons living below the poverty line. Education level reported the percentage of patients aged ≥25 years with at least a high school diploma. Residence was classified as a metro or nonmetro area according to the Rural‐Urban Continuum Code 2013. Median household income, poverty rate, unemployment rate, and education level were converted into categorical variables according to the interquartile ranges.

The outcomes of this study were breast cancer–specific survival (BCSS) and overall survival (OS). Breast cancer–specific survival was measured as the time from the date of diagnosis to the date of death attributed to breast cancer, date of last follow‐up, or December 31, 2016. Deaths caused by other factors were viewed as competing risks. The cumulative incidence function (CIF) was used to evaluate the likelihood of death. Gray's test was applied to find the difference in CIF among groups.^15^ The competing risks model was built based on the subdistribution analysis of competing risks.^16^ In the Cox regression model analyzing disease‐specific regression, patients who died from reasons other than breast cancer were defined as censored at the date of the last follow‐up. Overall survival was calculated as the time from the breast cancer diagnosis to death due to any cause, the date of last follow‐up, or December 31, 2016.

### 2.3 Construction of the nomograms

We determined the univariate prognostic factors of BCSS and OS using the Gray's test and log‐rank tests, respectively.^17^ Variables with *P* < .05 were entered into the multivariable Cox proportional hazards model. The final model selection was determined using a backward stepdown selection process based on the Akaike information criterion.^18^ The independent prognostic factors determined by the multivariate analysis were used to construct nomograms for BCSS and OS.

### 2.4 Validation and calibration of the nomograms

The nomograms were subjected to 1000 bootstrap resamples for validation. The concordance index (C‐index) was used to assess the discrimination performance of the nomograms.^19^ The value of the C‐index ranges from 0.5 to 1.0, with a higher c‐index indicating a better capacity to separate patients with different survival outcomes. We utilized previously introduced methods to compare the C‐index between two different models.^20^ The TNM staging system in this study is determined as the model including tumor size, number of positive lymph nodes, and metastasis. Calibration represents the capacity of a model to make accurate estimates of outcome. The observed rates vs the nomogram‐predicted probabilities of the models were used to construct calibration curves. In a well‐calibrated model, the predictions are expected to fall on a 45° diagonal line.

### 2.5 Statistical analysis

All statistical analyses were performed using R software, version 3.5.0 (http://www.r-project.org) and SPSS software, version 22.0 (SPSS Inc). The R packages cmprsk^21^ and rms^22^ were used for modeling and developing the nomograms. The rcorrp.cens function in the R package Hmisc^23^ was used for comparing the C‐index between two nomograms. Two‐sided *P* values less than .05 were considered statistically significant.

## 3 RESULTS

### 3.1 Characteristics of patients with different insurance statuses

We included 207 749 female patients who were diagnosed with malignant breast cancer during 2007‐2012 and had their race, county of residence, marital status, and insurance status recorded in the SEER database (Figure S1). The demographic and clinical characteristics of the cohort are summarized in Table 1. In this cohort, 182 552 patients had non‐Medicaid insurance, 21 935 had Medicaid coverage, and 3262 were uninsured when diagnosed with breast cancer. In the non‐Medicaid insured cohort, non‐Hispanic white patients accounted for 74.1%, which was higher than the percentage of the Medicaid (44.6%) and uninsured (46.0%) cohorts. Young patients who were uninsured or who had insurance through Medicaid accounted for 2.5‐fold or 1.8‐fold larger population, respectively, of being diagnosed with breast cancer compared with privately insured young patients; the fold change also appeared in the group aged 36‐50, but tended to shrink. The marital status data indicated that there were 124 832 married patients (60.1%), 29 955 single patients (14.4%), and 52 962 (25.5%) patients who were separated, divorced, or widowed. We collected and analyzed county‐level data of household income, poverty, employment, and education and found that non‐Medicaid insured patients lived in counties with a higher median household income ($63 340), lower poverty rate (13.1%), higher education level (87.5%), and lower unemployment rate (6.9%). More patients in the non‐Medicaid insured group are white, married, and living in a metro area. Medicaid patients seemed to reside in counties with lower median household income ($61 020, *P* < .001), higher poverty rates (16.7%, *P* < .001), higher unemployment rates (7.6%, *P* < .001), and lower education levels (87.4%, *P* < .001).

**Table 1:** Demographic and clinicopathological characteristics of breast cancer patients according to the insurance status at diagnosis

Characteristics	Non‐Medicaid insured	Medicaid	Uninsured	Total	*P* value^a^
Total	182 552 (87.9%)	21 935 (10.6%)	3262 (1.6%)	207 749 (100%)	
Age at diagnosis					<.001
18‐35	4331 (2.4%)	968 (4.4%)	192 (5.9%)	5491 (2.6%)	
36‐50	43 452 (23.8%)	6707 (30.6%)	1246 (38.2%)	51 405 (24.7%)	
51‐65	72 611 (39.8%)	9299 (42.4%)	1583 (48.5%)	83 493 (40.2%)	
>65	62 158 (34.0%)	4961 (22.6%)	241 (7.4%)	67 360 (32.4%)	
Race					<.001
NHW	135 361 (74.1%)	9788 (44.6%)	1499 (46.0%)	146 648 (70.6%)	
NHB	16 805 (9.2%)	4173 (19.0%)	706 (21.6%)	21 684 (10.4%)	
NHA	15 191 (8.3%)	2553 (11.6%)	279 (8.6%)	18 023 (8.7%)	
Hispanic	15 195 (8.3%)	5421 (24.7%)	778 (23.9%)	21 394 (10.3%)	
Histology					<.001
IDC	138 875 (76.1%)	17 358 (79.1%)	2608 (80.0%)	158 841 (76.5%)	
ILC	15 024 (8.2%)	1384 (6.3%)	185 (5.7%)	16 593 (8.0%)	
Others^b^	28 653 (15.7%)	3193 (14.6%)	469 (14.4%)	32 315 (15.6%）	
Grade					<.001
I	42 603 (23.3%)	3665 (16.7%)	470 (14.4%)	46 728 (22.5%)	
II	79 809 (43.7%)	8876 (40.5%)	1301 (39.9%)	89 986 (43.3%)	
III	60 140 (32.9%)	9404 (42.9%)	1491 (45.7%)	71 035 (34.2%)	
Tumor size (cm)					<.001
≤2	116 155 (63.6%)	10 322 (47.1%)	1518 (46.5%)	127 995 (61.6%)	
2‐5	55 389 (30.3%)	9025 (41.1%)	1315 (40.3%)	65 729 (31.6%)	
>5	11 008 (6.0%)	2588 (11.8%)	429 (13.2%)	14 025 (6.8%)	
No. of positive LNs					<.001
0	125 416 (68.7%)	12 607 (57.5%)	1855 (56.9%)	139 878 (67.3%)	
1‐3	40,274 (22.1%)	5821 (26.5%)	867 (26.6%)	46 962 (22.6%)	
4‐9	11 425 (6.3%)	2271 (10.4%)	372 (11.4%)	14 068 (6.8%)	
≥10	5437 (3.0%)	1236 (5.6%)	168 (5.2%)	6841 (3.3%)	
Metastasis					<.001
No	179 754 (98.5%)	21 278 (97.0%)	3174 (97.3%)	204 206 (98.3%)	
Yes	2798 (1.5%)	657 (3.0%)	88 (2.7%)	3543 (1.7%)	
ER status					<.001
Negative	32 263 (17.7%)	5067 (23.1%)	851 (26.1%)	38 181 (18.4%)	
Positive	150 289 (82.3%)	16 868 (76.9%)	2411 (73.9%)	169 568 (81.6%)	
PR status					<.001
Negative	51 471 (28.2%)	7437 (33.9%)	1173 (36.0%)	60 081 (28.9%)	
Positive	131 081 (71.8%)	14 498 (66.1%)	2089 (64.0%)	147 668 (71.1%)	
Surgery					<.001
No	901 (0.5%)	283 (1.3%)	59 (1.8%)	1243 (0.6%)	
BCS	104 969 (57.5%)	10 242 (46.7%)	1497 (46.7%)	116 708 (56.2%)	
Mastectomy	76 682 (42.0%)	11 410 (52.0%)	1706 (52.0%)	89 798 (43.2%)	
Chemotherapy					<.001
No/unknown	103 421 (56.7%)	10 062 (45.9%)	1227 (37.6%)	114 710 (55.2%)	
Yes	79 131 (43.3%)	11 873 (54.1%)	2035 (62.4%)	93 039 (44.8%)	
Radiation					<.001
No/unknown	80 176 (43.9%)	10 712 (48.8%)	1572 (48.2%)	92 460 (44.5%)	
Yes	102 376 (56.1%)	11 223 (51.2%)	1690 (51.8%)	115 289 (55.5%)	
Marital status					<.001
Married	115 585 (63.3%)	7778 (35.5%)	1469 (45.0%)	124 832 (60.1%)	
Single	22 472 (12.3%)	6473 (29.5%)	1010 (31.0%)	29 955 (14.4%)	
Separated/divorced/widowed	44 495 (24.4%)	7684 (35.0%)	783 (24.0%)	52 962 (25.5%)	
Residence^c^					<.001
Nonmetro area	17 291 (9.5%)	2801 (12.8%)	444 (13.6%)	20 536 (9.9%)	
Metro area	165 261 (90.5%)	19,134 (87.2%)	2818 (86.4%)	187 213 (90.1%)	
Median household income, US $^c^	63 340	61 020	61 020	62 330	<.001
Poverty rate^c^	13.1%	16.7%	16.3%	13.3%	<.001
Unemployment rate^c^	6.9%	7.6%	7.1%	6.9%	<.001
Education level^c^	87.5%	87.4%	86.5%	87.5%	<.001

Abbreviations: BCS, breast conserving surgery; ER, estrogen receptor; IDC, infiltrating ductal carcinoma; ILC, infiltrating lobular carcinoma; LN, lymph node; NHA, Non‐Hispanic Asian or Pacific Islander and American Indian/Alaska Native; NHB, Non‐Hispanic Black; NHW, Non‐Hispanic White; PR, progesterone receptor.

^a^ The chi‐square test for categorical variables and the Kruskal‐Wallis test for continuous variables were used to calculate *P* value.

^b^ Including other histology of invasive breast cancer except IDC and ILC.

^c^ All data are county level. Education level represented the percentage of patients aged ≥25 y with at least a high school diploma.

### 3.2 Impacts of age at diagnosis on survival in the uninsured, medicaid, and privately insured groups

**Figure 1:**
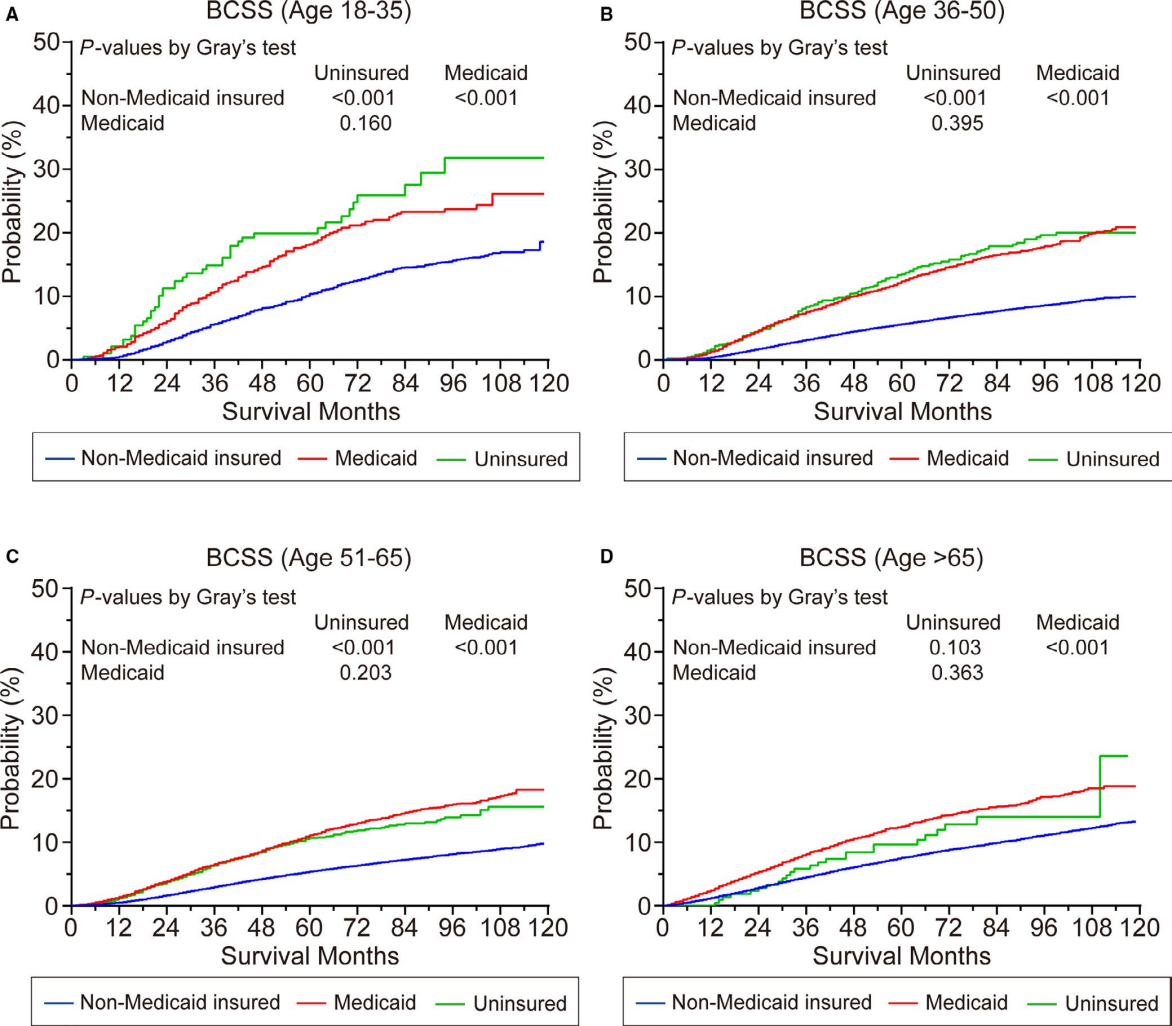
Breast cancer–specific survival (BCSS) of patients who were (A) 18‐35 y old, (B) 36‐50 y old, (C) 50‐65 y old, and (D) older than 65 y according to the insurance status at diagnosis. Among patients who were over 18 y old, BCSS was worse among Medicaid or uninsured patients vs those who were Non‐Medicaid insured (*P* < .001) except for the patients who were older than 65 y. Among patients who were older than 65 y, there was no significant difference in survival between patients with Non‐Medicaid insurance or without insurance

The median follow‐up of our identified cohort was 74 months. In all age groups, BCSS and OS of non‐Medicaid insured patients were better than uninsured patients and patients in Medicaid, except for the patients older than 65 years old (*P* < .001; Figure 1; Figure S2). There were no significant differences in BCSS between the Medicaid and uninsured cohorts. For patients aged 50‐64 years and older than 65 years at diagnosis, patients with Medicaid were associated with a worse OS than uninsured patients (*P* < .01; Figure S2C,D).

### 3.3 Influence of socioeconomic factors on patient outcomes

All the socioeconomic factors included in this study were confirmed to have significance for BCSS and OS through univariate analysis (Tables S1 and S2, respectively). The results of multivariate Cox regression models were shown in Table 2. Patients aged 36‐50 years at diagnosis had the most favorable survival compared with patients aged 18‐35 or older than 65 years (*P* < .001). Non‐Hispanic black patients experienced the worst survival compared with patients of other races (*P* < .001). As expected, patients with higher histological grade, larger tumors, more positive lymph nodes, metastasis, ER‐negative tumors, or PR‐negative tumors had worse BCSS and OS. Any treatment including surgery, chemotherapy, and radiation therapy decreased the cause‐specific risk of death and the overall risk of death. Interestingly, married (vs single vs separated/divorced/widowed; *P* < .001) and non‐Medicaid insured (vs Medicaid vs uninsured; *P* < .001) patients had a better prognosis. Furthermore, living in counties in the highest median household income quartile had favorable impacts on BCSS (HR = 0.838, 95% CI = 0.769‐0.913, *P* < .001) and OS (HR = 0.785, 95% CI = 0.738‐0.835, *P* < .001). Based on the multivariate analysis, there was no significant survival difference between patients who lived in counties with different place of residence, poverty level, or unemployment rate.

**Table 2:** Multivariate cox regression model of breast cancer–specific survival and overall survival among breast cancer patients

Characteristics	BCSS		OS	
	HR (95% CI)	*P* value	HR (95% CI)	*P* value
Age at diagnosis				
18‐35	Reference	—	Reference	—
36‐50	0.849 (0.787‐0.916)	<.001	0.796 (0.741‐0.855)	<.001
51‐65	0.897 (0.832‐0.967)	.004	0.980 (0.913‐1.051)	.569
>65	1.362 (1.261‐1.473)	<.001	2.391 (2.228‐2.566)	<.001
Race/ethnicity				
NHW	Reference	—	Reference	—
NHB	1.222 (1.170‐1.276)	<.001	1.144 (1.106‐1.182)	<.001
NHA	0.770 (0.723‐0.820)	<.001	0.748 (0.713‐0.785)	<.001
Hispanic	0.927 (0.881‐0.975)	.003	0.877 (0.843‐0.913)	<.001
Histology				
IDC	Reference	—	Reference	—
ILC	1.032 (0.973‐1.095)	.291	0.929 (0.892‐0.968)	<.001
Others^a^	0.925 (0.886‐0.967)	.001	0.953 (0.924‐0.982)	.002
Grade				
I	Reference	—	Reference	—
II	1.838 (1.719‐1.965)	<.001	1.185 (1.146‐1.226)	<.001
III	2.906 (2.713‐3.114)	<.001	1.572 (1.515‐1.632)	<.001
Tumor size (cm)				
≤2	Reference	—	Reference	—
2‐5	2.035 (1.958‐2.115)	<.001	1.619 (1.578‐1.662)	<.001
>5	3.159 (3.006‐3.320)	<.001	2.443 (2.351‐2.538)	<.001
Number of positive LNs				
0	Reference	—	Reference	—
1‐3	2.126 (2.044‐2.212)	<.001	1.575 (1.532‐1.620)	<.001
4‐9	3.832 (3.657‐4.015)	<.001	2.622 (2.527‐2.720)	<.001
≥10	5.344 (5.073‐5.630)	<.001	3.567 (3.418‐3.722)	<.001
Metastasis				
No	Reference	—	Reference	—
Yes	3.872 (3.686‐4.067)	<.001	3.074 (2.937‐3.216)	<.001
ER status				
Negative	Reference	—	Reference	—
Positive	0.719 (0.688‐0.751)	<.001	0.769 (0.743‐0.796)	<.001
PR status				
Negative	Reference	—	Reference	—
Positive	0.617 (0.592‐0.643)	<.001	0.745 (0.723‐0.769)	<.001
Surgery				
No	Reference	—	Reference	—
BCS	0.368 (0.332‐0.407)	<.001	0.440 (0.402‐0.481)	<.001
Mastectomy	0.442 (0.401‐0.487)	<.001	0.489 (0.448‐0.533)	<.001
Chemotherapy				
No/unknown	Reference	—	Reference	—
Yes	0.840 (0.809‐0.871)	<.001	0.668 (0.650‐0.686)	<.001
Radiation				
No/unknown	Reference	—	Reference	—
Yes	0.880 (0.851‐0.909)	<.001	0.792 (0.773‐0.812)	<.001
Marital status				
Married	Reference	—	Reference	—
Single	1.160 (1.111‐1.210)	<.001	1.252 (1.211‐1.295)	<.001
Separated/divorced/widowed	1.236 (1.193‐1.281)	<.001	1.449 (1.413‐1.485)	<.001
Insurance				
Non‐Medicaid insured	Reference	—	Reference	—
Medicaid	1.253 (1.201‐1.308)	<.001	1.403 (1.359‐1.449)	<.001
Uninsured	1.274 (1.156‐1.405)	<.001	1.334 (1.227‐1.451)	<.001
Residence^b^				
Nonmetro area	Reference	—	Reference	—
Metro area	0.978 (0.926‐1.033)	.434	0.977 (0.941‐1.016)	.243
Median household income^b^				
≤Quartile 1 (US $54 350)	Reference	—	Reference	—
≤Quartile 2 (US $62 330)	0.981 (0.929‐1.035)	.485	0.915 (0.879‐0.952)	<.001
≤Quartile 3 (US $78 020)	0.921 (0.856‐0.991)	.028	0.876 (0.831‐0.924)	<.001
>Quartile 3 (US $78 020)	0.838 (0.769‐0.913)	<.001	0.785 (0.738‐0.835)	<.001
Poverty rate^b^				
≤Quartile 1 (10.18%)	Reference	—	Reference	—
≤Quartile 2 (13.33%)	1.007 (0.945‐1.073)	.820	1.024 (0.979‐1.072)	.299
≤Quartile 3 (16.96%)	1.002 (0.916‐1.096)	.971	0.996 (0.934‐1.063)	.906
>Quartile 3 (16.96%)	0.977 (0.886‐1.078)	.646	0.990 (0.923‐1.063)	.790
Unemployment rate^b^				
≤Quartile 1 (5.68%)	Reference	—	Reference	—
≤Quartile 2 (6.91%)	0.974 (0.926‐1.025)	.315	0.970 (0.936‐1.006)	.103
≤Quartile 3 (7.80%)	0.940 (0.890‐0.993)	.026	0.929 (0.893‐0.966)	<.001
>Quartile 3 (7.80%)	0.986 (0.930‐1.045)	.627	1.011 (0.969‐1.054)	.615
Education level^b^				
≤Quartile 1 (82.88%)	Reference	—	Reference	—
≤Quartile 2 (87.54%)	1.003 (0.956‐1.051)	.912	1.022 (0.987‐1.057)	.222
≤Quartile 3 (91.08%)	0.963 (0.914‐1.015)	.162	0.989 (0.952‐1.027)	.554
>Quartile 3 (91.08%)	0.916 (0.859‐0.977)	.008	0.991 (0.946‐1.038)	.703

Abbreviations: BCS, breast conserving surgery; BCSS, breast cancer–specific survival; CI, confidence interval; ER, estrogen receptor; HR hazard ratio; IDC, infiltrating ductal carcinoma; ILC, infiltrating lobular carcinoma; LN, lymph node; NHA, Non‐Hispanic Asian or Pacific Islander and American Indian/Alaska Native; OS, overall survival; PR, progesterone receptor.

^a^ Including other histology of invasive breast cancer except IDC and ILC.

^b^ All data are county level. Education level represented the percentage of patients aged ≥25 y with at least a high school diploma.

### 3.4 Construction and validation of nomograms for BCSS and OS

**Figure 2:**
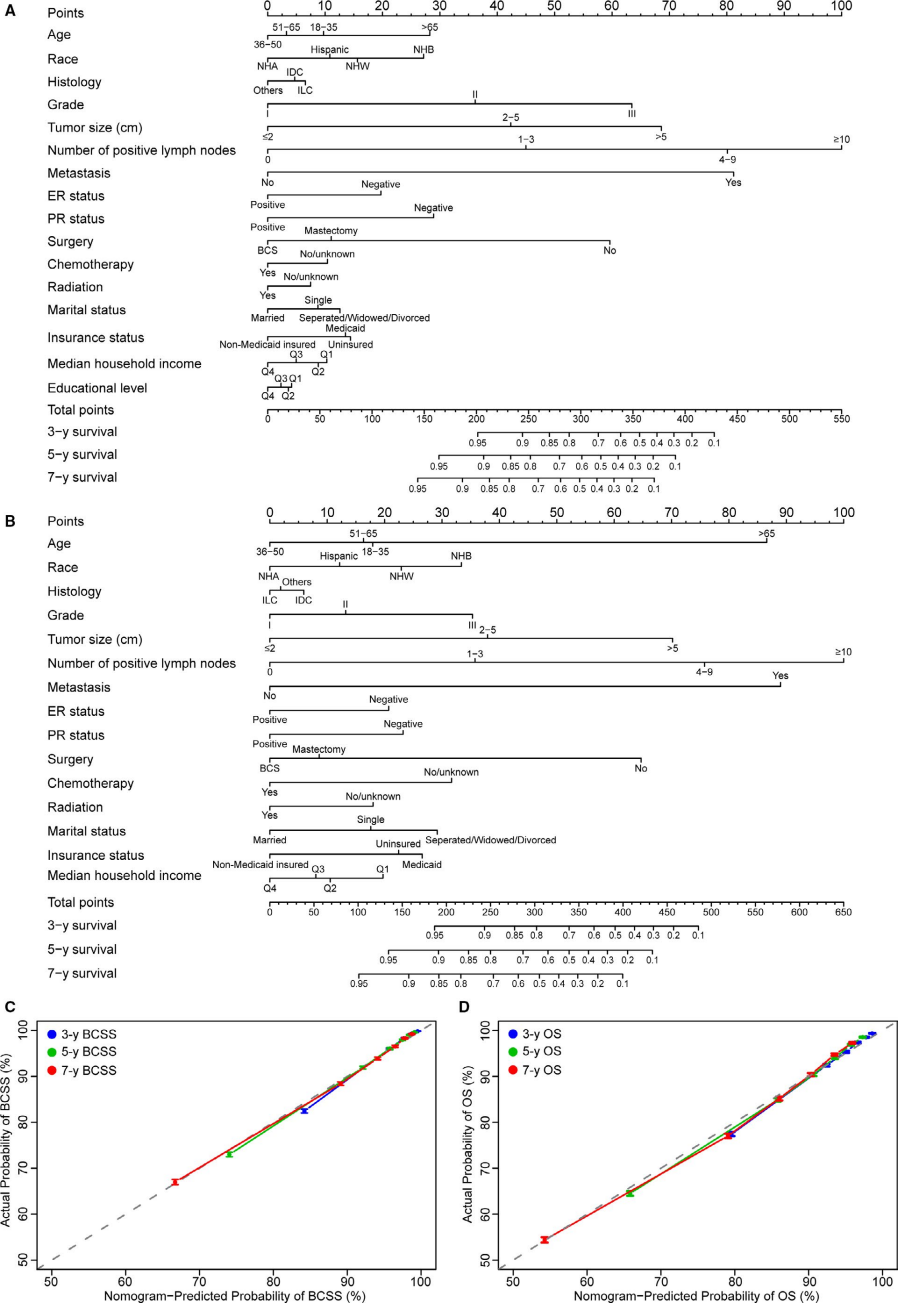
Prognostic nomograms (A, B) and calibration plots of survival probabilities at 3/5/7 y (C, D) in patients with breast cancer. Nomogram and calibration plots for BCSS (A, C). Nomogram and calibration plots for overall survival (OS) (B, D). Points of each variable can be estimated by drawing an upward vertical straight line from the variable value of the patient to the axis at the top flagged as “Points.” A vertical straights line is draw downward from sum of all variable values on the axis of “Total points” to calculate 3‐, 5‐, and 7‐y BCSS or OS. In calibration plots, actual survival is plotted on the vertical axis and predicted survival is plotted on the horizontal. Dotted grey line represents the ideal calibration model in which the predict survival is identical to the actual survival. Vertical bars represent 95% confidence intervals. BCS, breast conserving surgery; ER, estrogen receptor; IDC, infiltrating ductal carcinoma; ILC, infiltrating lobular carcinoma; NHA, Non‐Hispanic Asian or Pacific Islander and American Indian/Alaska Native; NHB, Non‐Hispanic Black; NHW, Non‐Hispanic White; PR, progesterone receptor

Nomograms including significant prognostic variables for BCSS and OS of breast cancer patients at 3‐, 5‐, and 7‐ years are presented in Figure 2A,B. Points in the nomograms are assigned based on the hierarchy of effects on survival and point assignment was listed in Table S3. The highest points are assigned to the number of positive lymph nodes in the nomogram for both BCSS and OS. Although histological variables and surgical procedures shared the largest contribution to the prognosis, socioeconomic variables, such as insurance status and marital status, moderately impacted the prognosis, while the level of median household income and education level played minor roles (Figure 2A,B). Calibration plots revealed high consistency between predicted and actual observed 3‐, 5‐, and 7‐year BCSS and OS for breast cancer patients (Figure 2C,D). The C‐indexes for the final nomograms for BCSS and OS were higher than those for the TNM staging system (0.835 vs 0.782, *P* < .001; 0.773 vs 0.676, *P* < .001, respectively; Table 3). A lower C‐index was generated by the nomogram of OS, which excluded all socioeconomic factors: marital status, insurance status, and level of median household income (0.773 vs 0.766, *P* < .001; Table 3).

**Table 3:** Comparison of C‐indexes for the nomograms and TNM staging system in patients with breast cancer

Items	BCSS		OS	
	C‐index (95% CI)	*P* value	C‐index (95% CI)	*P* value
Nomogram 1	0.835 (0.832‐0.838)	Reference	0.773 (0.771‐0.776)	Reference
TNM staging system	0.782 (0.779‐0.786)	<.001	0.676 (0.673‐0.679)	<.001
Nomogram 2 (excluding race)	0.834 (0.831‐0.837)	.356	0.772 (0.769‐0.775)	.253
Nomogram 3 (excluding all clinicopathological factors)^†^	0.715 (0.711‐0.719)	<.001	0.701 (0.698‐0.703)	<.001
Nomogram 4 (excluding all therapy information) ^‡^	0.832 (0.830‐0.835)	.165	0.768 (0.766‐0.771)	.002
Nomogram 5 (excluding all socioeconomic factors) ^§^	0.832 (0.829‐0.835)	.148	0.766 (0.763‐0.769)	<.001

Abbreviations: BCSS, breast cancer‐specific survival; CI, confidence interval; ER, estrogen receptor; OS, overall survival; PR, progesterone receptor.

^†^Clinicopathological factors include histology, grade, tumor size, number of positive lymph nodes, ER status and PR status.

^‡^Therapy information include surgery, chemotherapy and radiation.

^§^Socioeconomic factors include marital status, insurance, residence, median household income and education level.

**Figure 3:**
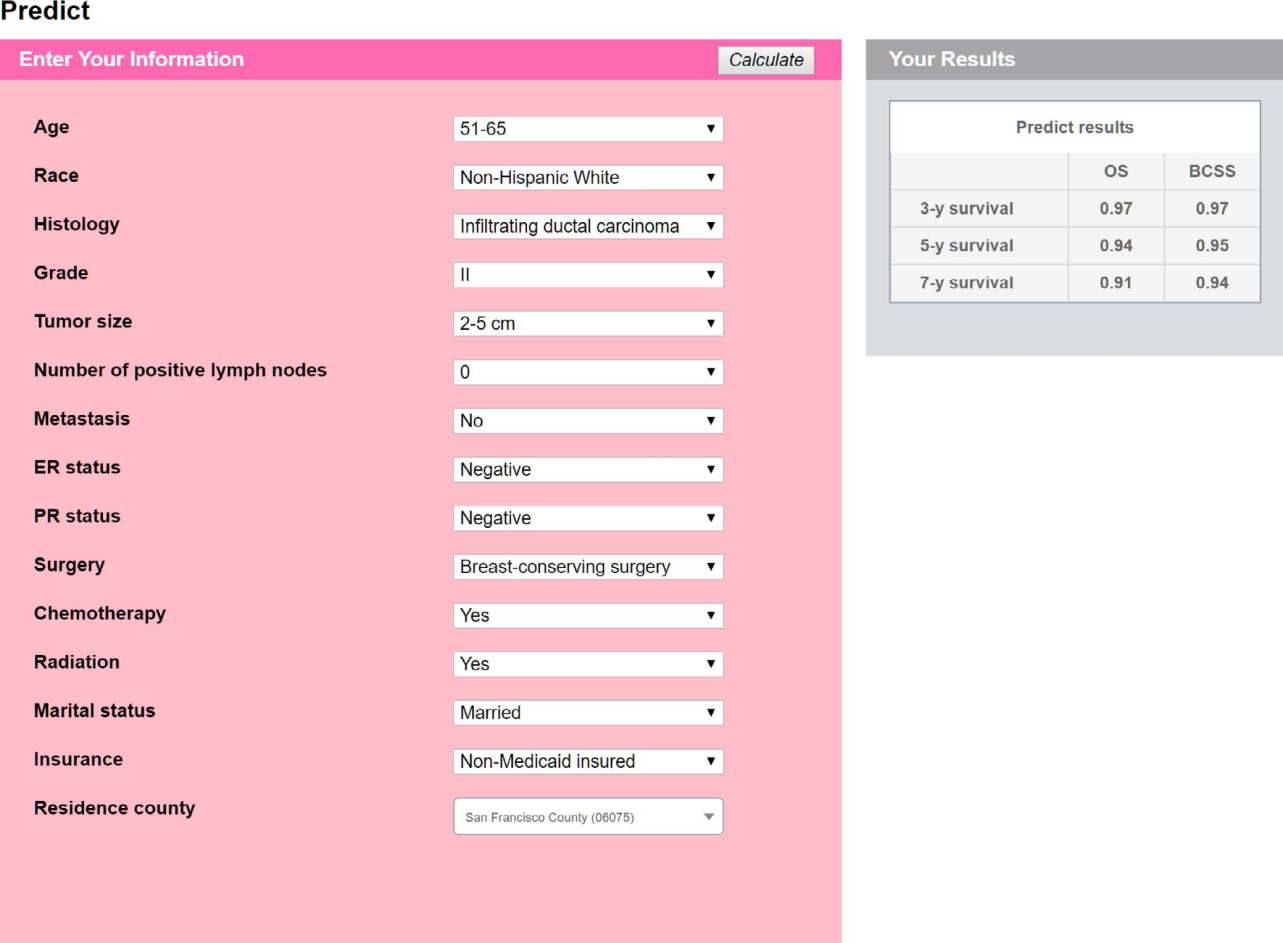
Screenshot from the web‐based nomograms, predicting 3‐, 5‐, and 7‐y BCSS/OS of imaginary patient. The nomograms are available at http://predictbcos.shaws.cn:8888. According to information of a patient, choose the value of each variable and then press the “Calculate” button

To improve the usability of these nomograms and the ability for doctors or patients to easily obtain results quickly and accurately, we transferred the data and formulas into a user‐friendly website. Figure 3 shows a snapshot of web‐based nomograms that are available on http://predictbcos.shaws.cn:8888. Visitors can predict survival of breast cancer as it relates to socioeconomic and clinicopathological factors by selecting values from drop‐down lists according to the individual situation and then clicking the button “Calculate”. For example, a 53‐year‐old white woman who was married and insured and lived in San Francisco had a 3‐cm, grade II, IDC tumor in her right breast. She then underwent breast conserving surgery and the pathological report showed that there was no lymph node metastasis or distant metastasis and the tumor was ER negative and PR negative. The patient also received chemotherapy and radiation. When we used the website to predict this woman's survival, we could find that the predicting results showed in the upper right corner that 3‐, 5‐, 7‐year BCSS rates were 0.97, 0.95, and 0.94, respectively, and 3‐, 5‐, 7‐year OS rates were 0.97, 0.94, and 0.91, respectively (Figure 3).

## 4 DISCUSSION

Unlike other causes of death, the morbidity and mortality of breast cancer show positive correlations with socioeconomic factors and vary substantially across countries and, with each county, are associated with the economic development, social factors, and lifestyles.^7^, ^8^, ^12^, ^13^, ^24^, ^25^, ^26^, ^27^, ^28^ A large number of population‐based retrospective studies have been conducted in many areas aimed to explore the association between breast cancer survival and socioeconomic factors.^7^, ^8^, ^13^, ^26^, ^27^, ^29^, ^30^, ^31^ To the best of our knowledge, this is the first attempt to develop web‐based nomograms that include socioeconomic factors for predicting BCSS and OS of patients with breast cancer. The user‐friendly website also provided convenience for using our models and obtaining more accurate results.

Our cohort was obtained from the SEER database and had a large sample size and wide distribution, which bolstered its representation of individuals with breast cancer. Through univariate analysis and subsequent multivariate analysis, we identified 16 and 15 variables including demographic, clinical, pathological, and socioeconomic factors as independent prognostic factors of BCSS and OS, respectively. In our study, marital status and insurance status were individual socioeconomic factors, whereas median household income level and educational level were area‐specific socioeconomic factors. Hence, only 15 questions were selected for our online prognostic tool (Figure 3), whereas the influence of two area‐level factors could be decided by the last question: county of residence. In this way, we could balance the credibility and simplicity of our models and avoid tedious evaluations of individual information. We also included place of residence, county‐level poverty rate, and unemployment rate, but these variables did not appear to significantly influence survival after the correction of confounders.

Survival inequality caused by socioeconomic factors, namely, insurance, marriage, income, region, and education, has been well documented for many cancer types in the literature.^2^, ^10^, ^32^, ^33^ Obviously, insurance directly effects patients’ access to healthcare, either via screening for early diseases or persisting to the conclusion of treatments.^33^ Although marriage seems to be a more protective factor for males than for females, our results were in alignment with previous studies that married women have more favorable survival.^10^, ^11^, ^25^ Investments in the healthcare system and cancer treatment largely depend on the economic power of a country or a state, which is closely correlated with medical level and inclusion in universal health coverage.^34^ Different states have an uneven distribution of medical resources and different policies concerning breast cancer screening in women, leading to regional disparities in early diagnosis and use of effective treatments. The educational level of women impacts their opinions toward mammogram screening and concerns after a cancer diagnosis.^5^

In many states in the United States, surgeries and adjuvant systemic treatments of breast cancer are fully reimbursed by all types of health insurance. Among newly diagnosed breast cancer in 18 SEER registries, insurance status showed effects on stages at diagnosis, whereas young patients showed different proportions when populations were grouped by insurance status (Table 1). In addition to the effects on diagnosis, uninsured or Medicaid‐insured statuses were indicated as unfavorable factors for BCSS and OS compared with privately insured patients among all age groups, which was consistent with a previous population‐based study (Figure 1).^35^ According to our data, uninsured patients were more likely to “die by breast cancer”, with 7.6% 3‐year and 12.2% 5‐year cumulative incidences of death resulting from breast cancer, whereas the 3‐ and 5‐year cumulative incidences of death resulting from other reasons were 1.6% and 3.1%, respectively (Table S1). There are various types of insurance in the United States, including private insurance through employers or directly purchased and Medicaid or Medicare insurance provided by the government, but some people still lack any coverage, especially young adults. In 2016, while the percentage of uninsured people was 8.8% of the population in the United States, the peak uninsured rate occurred in young adults aged 26‐34 (15.7%), followed by the population aged 19‐25 (13.1%).^36^ Since the Patient Protection and Affordable Care Act (ACA) passed in 2010, under policies of the ACA, young people under age 26 can remain on their parents' insurance plan, which resulted in a rise in health coverage of young people aged 19‐26.^36^ However, people between the ages of 26 and 34, who are students or at the beginning of their careers, need more support for insurance coverage, and these demands affect not only breast cancer but also other cancers and hematologic malignancies, as shown in several previous studies.^29^, ^37^, ^38^ The delays in diagnosis and treatments related to insurance, which may contribute to a poor prognosis, breast cancer in younger patients intrinsically exhibits more aggressive biological behaviors.^24^ In addition, the preservation of fertility for young patients also leads to greater medical expenses, and expenses associated with long‐term follow‐up can also limit adherence. The association between insurance status and breast cancer may not be generalizable to the entire world due to the diversity in the healthcare systems of different countries.

Marital status is an integral part of the socioeconomic status, and many lines of evidence suggest that it can affect the risk of breast cancer, acceptance of breast cancer screening, stage at diagnosis, and adherence to treatment, follow‐up, and survival throughout one's lifetime. In a recent study of Palestinian people, women mentioned a series of barriers in preventing them from having a mammogram, such as shyness, fear of being diagnosed, being busy with children, and anxiety regarding marriage stability.^5^ With respect to survival, our study showed that marriage was a protective factor in the treatment of cancers (Table 2; Table S1), which was consistent with the conclusions of Aizer's study of the 10 most clinically significant cancers affecting Americans.^10^ In our nomograms, marital status was weighted heavier than insurance status in the model of predicting the OS of breast cancer; conversely, insurance outweighed marital status in predicting BCSS (Figure 2). A link between marital status and insurance status was that many people obtained health insurance through their spouse. According to the results of our analysis, the uninsured rate was highest among single patients, and the insured rate was highest among married patients from 2007 to 2012 (Table 1), and based on data from 2016, the uninsured rate of separated people was also approximately 10 percentage points higher than that among people who were married.^35^ Herein, marital status not only directly affected survival of patients with breast cancer but also indirectly affected survival through impacts on insurance status. After a diagnosis of breast cancer, depression and anxiety are common mentalities for most patients. Although some married patients can obtain support from their spouse, people who are single, separated, divorced, or widowed might have to face their situation alone, increasing the risk of nonadherence. Marriage is essentially a kind of social support, and psycho‐oncology services are warranted to improve prognosis by reducing worries and isolation among all patients and their families, especially for single, separated, divorced, or widowed patients.^39^

County‐level assessments of median household income and education indirectly reflect individual levels, and assessments at this level make sense because the county is the smallest geographic unit in policy legislations. In addition, county‐level variables mainly embody socioeconomic inequalities between different areas. In these modern times, the behaviors and dietary habits that increase breast cancer incidence, such as less physical activity, radiation exposure, smoking habits, environmental pollution, and high‐fat diets, are more common in residents of low‐income areas than in residents of high‐income areas.^40^ Individual income level directly affects individual insurance status. According to the annual report on health insurance coverage in the United States, people with a higher household income level had a higher overall health insurance coverage rate than people with a lower household income level, and lower‐income populations showed an increasing dependency on insurance coverage offered by the government.^35^ County‐level income represents the economic strength of the whole area. Weakness in financial strength may be related to fewer cancer screening programs,^41^ later stage of diagnosis,^34^ and lower likelihood of optimal treatment.^42^

Education seemed to be a controversial factor in the occurrence, diagnosis, and survival of breast cancer. With the respect to morbidity, studies in the literature have indicated that greater education led to a higher risk of developing breast cancer in women.^26^ In contrast, cervical cancer, similar to breast cancer as one of the most common cancers diagnosed in women, presented different patterns when exploring educational impacts on tumor incidence, and women who received more than 12 years of education had a sharp reduction in the occurrence of cervical cancer, which might be associated with differences in the pathogenesis of different cancers.^41^ Higher education tends to be associated with less manual labor (fewer physical activities), stressful work, late age at first birth, and null parity, factors known to increase the incidence of breast cancer, whereas cervical cancer is largely caused by infection with human papillary virus, which might explain the difference between the impact of education on the incidence rates of these two cancers.^43^, ^44^ Interestingly, while education level could modify the effect of psychological distress on accepting screening for colorectal and lung cancers, it did not exhibit this effect on breast cancer screening in countries with low cancer screening rates.^45^ From the perspective of survival, our study supported education as a protective factor for BCSS and OS (Table 2), which was consistent with results of previous studies in which women with high education levels had better survival than women with low education levels, which suggests that education helps women understand the benefits of conducting breast self‐examination and accepting routine breast cancer screening programs, and it can improve patient access to effective treatments.^13^, ^26^ However, the findings are not entirely in accordance with this point. A national study from Belgium reported that higher‐educated women had higher mortality than women with less education among postmenopausal women, with no significant gap among premenopausal women.^44^ The divergence of conclusions may be related to the different dimensions of the data; that is, we used county‐ and state‐level metrics from SEER, and the study from Belgium used individual‐level data. Additionally, the populations in different countries may have completely different socioeconomic environments; furthermore, individual education level is a very stable factor and can be identified in adolescence, but the education level of the patient's spouse can also be taken into consideration.

Our findings should be interpreted within SEER registry areas, and the online calculator we provided can only be applied for breast cancer patients in SEER registries. Although robust and population based, our research still has some limitations. The complexity of individual socioeconomic information and barriers in access to data limited us from including all factors related in our study, and our data were mainly obtained from the SEER database. In addition, the healthcare insurance system in the United States is very complicated. There are many different types of insurance, varying from state to state, and this study population predates the ACA; thus, how these changes would affect our findings is not known. Moreover, although we included data related to chemotherapy and radiotherapy for analysis, these treatment factors had some biases according to the SEER database. We also lacked access to some variables, such as HER2 status, Ki‐67 positivity, dietary, behavior, and out‐of‐pocket cancer treatment expenses, and therefore could not investigate their association with survival. Due to the emergence of endocrine therapies and targeted therapies, the mortality of breast cancer decreased by 40% from 1989 to 2016,^46^, ^47^ but the data on adherence to these therapies cannot yet be acquired.

## CONFLICT OF INTEREST

None declared.

## AUTHORS' CONTRIBUTIONS

Conception and design: PJ, YG, CCJ, HX, GHD, and ZMS. Development of methodology: PJ, YG, CCJ, XH, and ZMS. Acquisition of data: PJ and YG. Analysis and interpretation of data: PJ, YG, XH, GHD, and ZMS. Writing, review, and/or revision of manuscript: PJ, YG, CCJ, HX, GHD, and ZMS. Study supervision: XH, GHD, and ZMS. All authors read and approved the final manuscript.

## Data Availability

The datasets generated and analyzed during this study are available from Surveillance, Epidemiology, and End Results (SEER) Program (http://www.seer.cancer.gov) SEER*Stat Database: Incidence—SEER 18 Regs Custom Data (with additional treatment fields), Nov 2018 Sub (1975‐2016 varying), National Cancer Institute, DCCPS, Surveillance Research Program, released April 2019, based on the November 2018 submission.
