# Supplementary figures and images for: Association between socioeconomic factors at diagnosis and survival in breast cancer: A population‐based study
Source: Cancer Med. 2020 Jan 20;9(5):1922–36. doi: 10.1002/cam4.2842 (PMC7050085; doi:10.1002/cam4.2842)

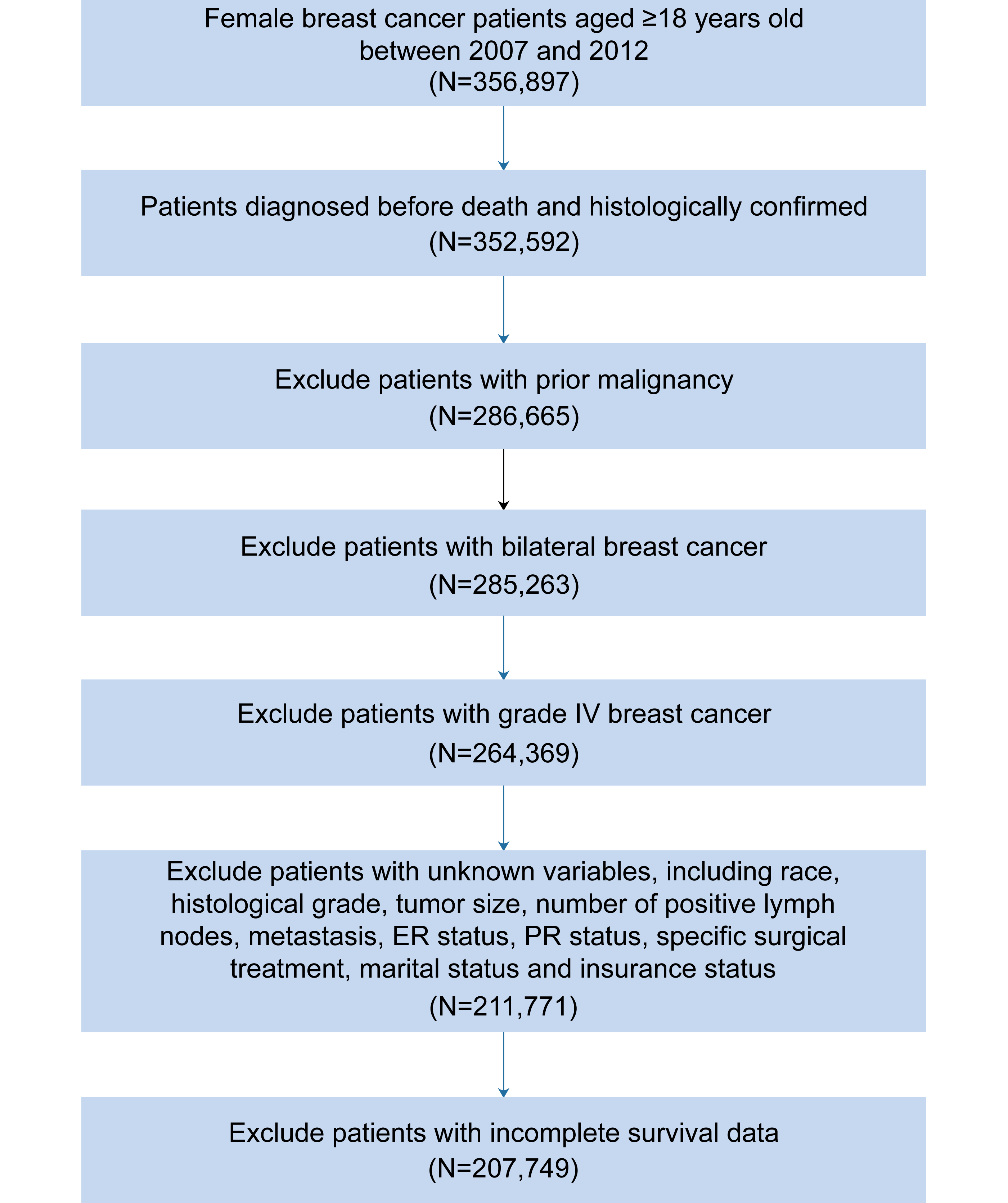

Supplement: Supplementary file 1 [file CAM4-9-1922-s001.tif]

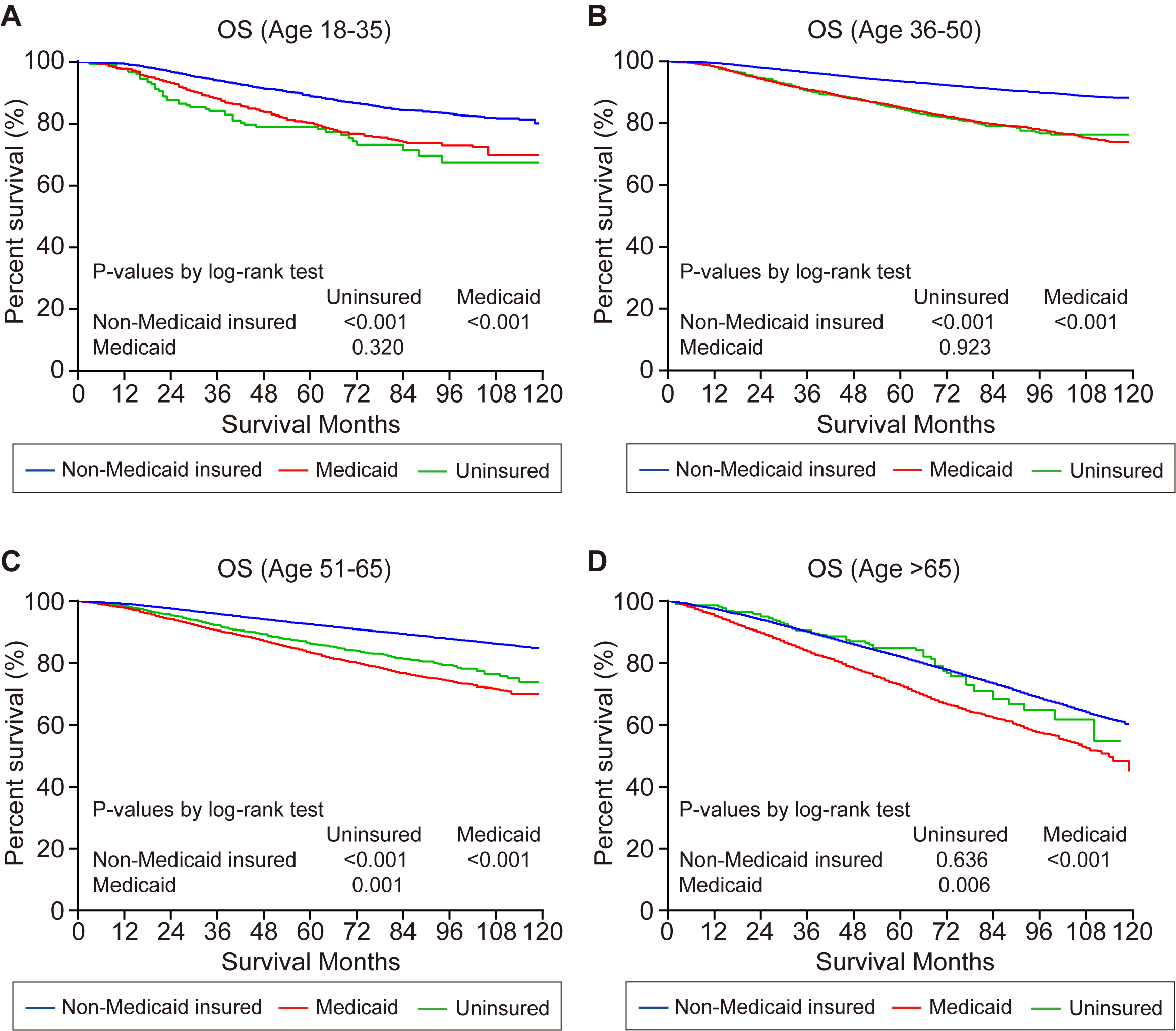

Supplement: Supplementary file 2 [file CAM4-9-1922-s002.tif]
