# Supplementary material for: Association between socioeconomic factors at diagnosis and survival in breast cancer: A population‐based study
Source: Cancer Med. 2020 Jan 20;9(5):1922–36. doi: 10.1002/cam4.2842 (PMC7050085; doi:10.1002/cam4.2842)
Supplement: Supplementary file 3 [file CAM4-9-1922-s003.docx]

| **Supplementary Table 1.** Three- and five-year cumulative incidences of death among breast cancer patients | | | | | | | |
| --- | --- | --- | --- | --- | --- | --- | --- |
| Characteristics | Cumulative Incidence of Death Resulting From Breast Cancer | | |  | Cumulative Incidence of Death Resulting From Other Causes | | |
|  | 3-year | 5-year | *P*-value |  | 3-year | 5-year | *P*-value |
| All patients | 0.041 | 0.070 |  |  | 0.026 | 0.052 |  |
| Age at diagnosis |  |  | <0.001 |  |  |  | <0.001 |
| 18-35 | 0.068 | 0.121 |  |  | 0.005 | 0.010 |  |
| 36-50 | 0.039 | 0.067 |  |  | 0.007 | 0.012 |  |
| 51-65 | 0.034 | 0.061 |  |  | 0.014 | 0.026 |  |
| > 65 | 0.048 | 0.079 |  |  | 0.059 | 0.117 |  |
| Race/ethnicity |  |  | <0.001 |  |  |  | <0.001 |
| NHW | 0.036 | 0.063 |  |  | 0.028 | 0.056 |  |
| NHB | 0.076 | 0.123 |  |  | 0.034 | 0.062 |  |
| NHA | 0.027 | 0.049 |  |  | 0.016 | 0.030 |  |
| Hispanic | 0.046 | 0.078 |  |  | 0.018 | 0.036 |  |
| Histology |  |  | <0.001 |  |  |  | <0.001 |
| IDC | 0.043 | 0.073 |  |  | 0.026 | 0.050 |  |
| ILC | 0.030 | 0.062 |  |  | 0.028 | 0.056 |  |
| Others^†^ | 0.036 | 0.060 |  |  | 0.028 | 0.057 |  |
| Grade |  |  | <0.001 |  |  |  | <0.001 |
| I | 0.007 | 0.016 |  |  | 0.024 | 0.049 |  |
| II | 0.023 | 0.047 |  |  | 0.026 | 0.053 |  |
| III | 0.086 | 0.135 |  |  | 0.029 | 0.052 |  |
| Tumor size (cm) |  |  | <0.001 |  |  |  | <0.001 |
| ≤ 2 | 0.014 | 0.027 |  |  | 0.022 | 0.045 |  |
| 2-5 | 0.064 | 0.113 |  |  | 0.034 | 0.062 |  |
| > 5 | 0.176 | 0.261 |  |  | 0.039 | 0.071 |  |
| Number of positive LNs |  |  | <0.001 |  |  |  | <0.001 |
| 0 | 0.018 | 0.032 |  |  | 0.024 | 0.050 |  |
| 1-3 | 0.056 | 0.097 |  |  | 0.028 | 0.052 |  |
| 4-9 | 0.131 | 0.217 |  |  | 0.036 | 0.063 |  |
| ≥ 10 | 0.224 | 0.352 |  |  | 0.046 | 0.082 |  |
| Metastasis |  |  | <0.001 |  |  |  | <0.001 |
| No | 0.035 | 0.061 |  |  | 0.026 | 0.052 |  |
| Yes | 0.395 | 0.552 |  |  | 0.048 | 0.088 |  |
| ER status |  |  | <0.001 |  |  |  | 0.043 |
| Negative | 0.113 | 0.162 |  |  | 0.032 | 0.056 |  |
| Positive | 0.024 | 0.049 |  |  | 0.025 | 0.051 |  |
| PR status |  |  | <0.001 |  |  |  | 0.003 |
| Negative | 0.092 | 0.137 |  |  | 0.031 | 0.057 |  |
| Positive | 0.020 | 0.042 |  |  | 0.025 | 0.050 |  |
| Surgery |  |  | <0.001 |  |  |  | <0.001 |
| No | 0.277 | 0.368 |  |  | 0.059 | 0.100 |  |
| BCS | 0.021 | 0.039 |  |  | 0.022 | 0.045 |  |
| Mastectomy | 0.064 | 0.107 |  |  | 0.032 | 0.062 |  |
| Chemotherapy |  |  | <0.001 |  |  |  | <0.001 |
| No/unknown | 0.025 | 0.043 |  |  | 0.036 | 0.071 |  |
| Yes | 0.060 | 0.102 |  |  | 0.015 | 0.028 |  |
| Radiation |  |  | <0.001 |  |  |  | <0.001 |
| No/unknown | 0.050 | 0.082 |  |  | 0.038 | 0.071 |  |
| Yes | 0.033 | 0.060 |  |  | 0.017 | 0.037 |  |
| Marital status |  |  | <0.001 |  |  |  | <0.001 |
| Married | 0.032 | 0.057 |  |  | 0.017 | 0.033 |  |
| Single | 0.052 | 0.087 |  |  | 0.024 | 0.045 |  |
| Separated/divorced/widowed | 0.055 | 0.089 |  |  | 0.051 | 0.101 |  |
| Insurance |  |  | <0.001 |  |  |  | <0.001 |
| Non-Medicaid insured | 0.036 | 0.063 |  |  | 0.025 | 0.050 |  |
| Medicaid | 0.074 | 0.121 |  |  | 0.039 | 0.075 |  |
| Uninsured | 0.076 | 0.122 |  |  | 0.016 | 0.031 |  |
| Residence^‡^ |  |  | <0.001 |  |  |  | <0.001 |
| Non-metro area | 0.048 | 0.082 |  |  | 0.037 | 0.072 |  |
| Metro area | 0.040 | 0.068 |  |  | 0.025 | 0.050 |  |
| Median household income^‡^ |  |  | <0.001 |  |  |  | <0.001 |
| ≤ Quartile 1 (US $54,350) | 0.052 | 0.086 |  |  | 0.036 | 0.070 |  |
| ≤ Quartile 2 (US $62,330) | 0.044 | 0.075 |  |  | 0.025 | 0.050 |  |
| ≤ Quartile 3 (US $78,020) | 0.037 | 0.064 |  |  | 0.024 | 0.048 |  |
| > Quartile 3 (US $78,020) | 0.030 | 0.054 |  |  | 0.021 | 0.040 |  |
| Poverty rate^‡^ |  |  | <0.001 |  |  |  | <0.001 |
| ≤ Quartile 1 (10.18%) | 0.031 | 0.055 |  |  | 0.021 | 0.042 |  |
| ≤ Quartile 2 (13.33%) | 0.037 | 0.064 |  |  | 0.024 | 0.049 |  |
| ≤ Quartile 3 (16.96%) | 0.045 | 0.076 |  |  | 0.027 | 0.053 |  |
| > Quartile 3 (16.96%) | 0.052 | 0.086 |  |  | 0.034 | 0.066 |  |
| Unemployment rate^‡^ |  |  | <0.001 |  |  |  | <0.001 |
| ≤ Quartile 1 (5.68%) | 0.033 | 0.059 |  |  | 0.024 | 0.048 |  |
| ≤ Quartile 2 (6.91%) | 0.039 | 0.066 |  |  | 0.026 | 0.050 |  |
| ≤ Quartile 3 (7.80%) | 0.042 | 0.072 |  |  | 0.023 | 0.048 |  |
| > Quartile 3 (7.80%) | 0.050 | 0.083 |  |  | 0.033 | 0.063 |  |
| Education level^‡^ |  |  | <0.001 |  |  |  | <0.001 |
| ≤ Quartile 1 (82.88%) | 0.048 | 0.082 |  |  | 0.029 | 0.057 |  |
| ≤ Quartile 2 (87.54%) | 0.044 | 0.075 |  |  | 0.028 | 0.054 |  |
| ≤ Quartile 3 (91.08%) | 0.037 | 0.064 |  |  | 0.025 | 0.049 |  |
| > Quartile 3 (91.08%) | 0.034 | 0.058 |  |  | 0.024 | 0.047 |  |
| Abbreviations: ER, estrogen receptor; IDC, infiltrating ductal carcinoma; ILC, infiltrating lobular carcinoma; LN, lymph node; NHA, Non-Hispanic Asian or Pacific Islander and American Indian/Alaska Native; PR, progesterone receptor.  ^†^ Including other histology of invasive breast cancer except IDC and ILC.  ^‡^ All data are county-level. Education level represented the percentage of patients aged ≥25 years with at least a high school diploma. | | | | | | | |

| **Supplementary Table 2.** Three-and five-year probability of overall survival among breast cancer patients | | | | |
| --- | --- | --- | --- | --- |
| Characteristics | Probability of overall survival | |  | P-value |
|  | 3-year | 5-year |  |  |
| All patients | 0.934 | 0.882 |  |  |
| Age at diagnosis |  |  |  | <0.001 |
| 18-35 | 0.926 | 0.870 |  |  |
| 36-50 | 0.955 | 0.922 |  |  |
| 51-65 | 0.952 | 0.914 |  |  |
| > 65 | 0.896 | 0.813 |  |  |
| Race |  |  |  | <0.001 |
| NHW | 0.937 | 0.885 |  |  |
| NHB | 0.893 | 0.822 |  |  |
| NHA | 0.958 | 0.923 |  |  |
| Hispanic | 0.938 | 0.888 |  |  |
| Histology |  |  |  | 0.004 |
| IDC | 0.932 | 0.881 |  |  |
| ILC | 0.943 | 0.885 |  |  |
| Others^†^ | 0.937 | 0.886 |  |  |
| Grade |  |  |  | <0.001 |
| I | 0.969 | 0.936 |  |  |
| II | 0.951 | 0.903 |  |  |
| III | 0.888 | 0.820 |  |  |
| Tumor size (cm) |  |  |  | <0.001 |
| ≤ 2 | 0.965 | 0.929 |  |  |
| 2-5 | 0.904 | 0.832 |  |  |
| > 5 | 0.792 | 0.686 |  |  |
| Number of positive LNs |  |  |  | <0.001 |
| 0 | 0.959 | 0.920 |  |  |
| 1-3 | 0.917 | 0.856 |  |  |
| 4-9 | 0.838 | 0.733 |  |  |
| ≥ 10 | 0.740 | 0.595 |  |  |
| Metastasis |  |  |  | <0.001 |
| No | 0.940 | 0.890 |  |  |
| Yes | 0.575 | 0.408 |  |  |
| ER status |  |  |  | <0.001 |
| Negative | 0.859 | 0.791 |  |  |
| Positive | 0.951 | 0.902 |  |  |
| PR status |  |  |  | <0.001 |
| Negative | 0.880 | 0.813 |  |  |
| Positive | 0.956 | 0.910 |  |  |
| Surgery |  |  |  | <0.001 |
| No | 0.681 | 0.569 |  |  |
| BCS | 0.958 | 0.918 |  |  |
| Mastectomy | 0.906 | 0.838 |  |  |
| Chemotherapy |  |  |  | 0.004 |
| No/unknown | 0.940 | 0.889 |  |  |
| Yes | 0.927 | 0.873 |  |  |
| Radiation |  |  |  | <0.001 |
| No/unknown | 0.913 | 0.853 |  |  |
| Yes | 0.950 | 0.904 |  |  |
| Marital status |  |  |  | <0.001 |
| Married | 0.952 | 0.911 |  |  |
| Single | 0.925 | 0.872 |  |  |
| Separated/divorced/widowed | 0.897 | 0.818 |  |  |
| Insurance |  |  |  | <0.001 |
| Non-Medicaid insured | 0.940 | 0.890 |  |  |
| Medicaid | 0.890 | 0.813 |  |  |
| Uninsured | 0.909 | 0.850 |  |  |
| Residence^‡^ |  |  |  | <0.001 |
| Non-metro area | 0.916 | 0.852 |  |  |
| Metro area | 0.936 | 0.885 |  |  |
| Median household income^‡^ |  |  |  | <0.001 |
| ≤ Quartile 1 (US $54,350) | 0.914 | 0.849 |  |  |
| ≤ Quartile 2 (US $62,330) | 0.932 | 0.879 |  |  |
| ≤ Quartile 3 (US $78,020) | 0.940 | 0.892 |  |  |
| > Quartile 3 (US $78,020) | 0.950 | 0.907 |  |  |
| Poverty rate^‡^ |  |  |  | <0.001 |
| ≤ Quartile 1 (10.18%) | 0.948 | 0.905 |  |  |
| ≤ Quartile 2 (13.33%) | 0.940 | 0.891 |  |  |
| ≤ Quartile 3 (16.96%) | 0.930 | 0.875 |  |  |
| > Quartile 3 (16.96%) | 0.916 | 0.853 |  |  |
| Unemployment rate^‡^ |  |  |  | <0.001 |
| ≤ Quartile 1 (5.68%) | 0.943 | 0.895 |  |  |
| ≤ Quartile 2 (6.91%) | 0.937 | 0.887 |  |  |
| ≤ Quartile 3 (7.80%) | 0.936 | 0.884 |  |  |
| > Quartile 3 (7.80%) | 0.919 | 0.859 |  |  |
| Education level^‡^ |  |  |  | <0.001 |
| ≤ Quartile 1 (82.88%) | 0.924 | 0.866 |  |  |
| ≤ Quartile 2 (87.54%) | 0.929 | 0.875 |  |  |
| ≤ Quartile 3 (91.08%) | 0.939 | 0.889 |  |  |
| > Quartile 3 (91.08%) | 0.943 | 0.897 |  |  |
| Abbreviations: BCS, breast conserving surgery; ER, estrogen receptor; IDC, infiltrating ductal carcinoma; ILC, infiltrating lobular carcinoma; LN, lymph node; NHA, Non-Hispanic Asian or Pacific Islander and American Indian/Alaska Native; NHB, Non-Hispanic Black; NHW, Non-Hispanic White; PR, progesterone receptor  ^†^ Including other histology of invasive breast cancer except IDC and ILC.  ^‡^ All data are county-level. Education level represented the percentage of patients aged ≥25 years with at least a high school diploma. | | | | |

| **Supplementary Table 3.** Point Assignment of Nomograms for OS and BCSS | | |
| --- | --- | --- |
| Characteristics and Prognostic Score | Score for BCSS | Score for OS |
| Age at diagnosis |  |  |
| 18-35 | 10 | 18 |
| 36-50 | 0 | 0 |
| 51-65 | 3 | 16 |
| > 65 | 28 | 87 |
| Race/ethnicity |  |  |
| NHW | 16 | 23 |
| NHB | 27 | 33 |
| NHA | 0 | 0 |
| Hispanic | 11 | 12 |
| Histology |  |  |
| IDC | 5 | 6 |
| ILC | 7 | 0 |
| Others^†^ | 0 | 2 |
| Grade |  |  |
| I | 0 | 0 |
| II | 36 | 13 |
| III | 63 | 35 |
| Tumor size (cm) |  |  |
| ≤ 2 | 0 | 0 |
| 2-5 | 42 | 38 |
| > 5 | 69 | 70 |
| Number of positive LNs |  |  |
| 0 | 0 | 0 |
| 1-3 | 45 | 36 |
| 4-9 | 80 | 76 |
| ≥ 10 | 100 | 100 |
| Metastasis |  |  |
| No | 0 | 0 |
| Yes | 81 | 89 |
| ER status |  |  |
| Negative | 20 | 21 |
| Positive | 0 | 0 |
| PR status |  |  |
| Negative | 29 | 23 |
| Positive | 0 | 0 |
| Surgery |  |  |
| No | 60 | 65 |
| BCS | 0 | 0 |
| Mastectomy | 11 | 9 |
| Chemotherapy |  |  |
| No/unknown | 10 | 32 |
| Yes | 0 | 0 |
| Radiation |  |  |
| No/unknown | 7 | 18 |
| Yes | 0 | 0 |
| Marital status |  |  |
| Married | 0 | 0 |
| Single | 9 | 18 |
| Separated/divorced/widowed | 13 | 29 |
| Insurance |  |  |
| Non-Medicaid insured | 0 | 0 |
| Medicaid | 14 | 27 |
| Uninsured | 14 | 22 |
| Median household income^‡^ |  |  |
| ≤ Quartile 1 (US $54,350) | 10 | 20 |
| ≤ Quartile 2 (US $62,330) | 9 | 11 |
| ≤ Quartile 3 (US $78,020) | 5 | 8 |
| > Quartile 3 (US $78,020) | 0 | 0 |
| Education level^‡^ |  |  |
| ≤ Quartile 1 (82.88%) | 4 | - |
| ≤ Quartile 2 (87.54%) | 4 | - |
| ≤ Quartile 3 (91.08%) | 2 | - |
| > Quartile 3 (91.08%) | 0 | - |
| Abbreviations: BCSS, breast cancer-specific survival; ER, estrogen receptor; IDC, infiltrating ductal carcinoma; ILC, infiltrating lobular carcinoma; LN, lymph node; NHA, Non-Hispanic Asian or Pacific Islander and American Indian/Alaska Native; OS, overall survival; PR, progesterone receptor.  ^†^ Including other histology of invasive breast cancer except IDC and ILC.  ^‡^ All data are county-level. Education level represented the percentage of patients aged ≥25 years with at least a high school diploma. | | |
